# Application of symmetry evaluation to deep learning algorithm in detection of mastoiditis on mastoid radiographs

**DOI:** 10.1038/s41598-023-32147-w

**Published:** 2023-04-01

**Authors:** Dongjun Choi, Leonard Sunwoo, Sung-Hye You, Kyong Joon Lee, Inseon Ryoo

**Affiliations:** 1 Department of Radiology, Seoul National University Bundang Hospital, Seongnam-si, Gyeonggi-do Korea; 2 Department of Radiology, Korea University Anam Hospital, Seoul, Korea; 3 Department of Radiology, Korea University Guro Hospital, Korea University College of Medicine, Seoul, Korea

**Keywords:** Infectious diseases, Medical research

## Abstract

As many human organs exist in pairs or have symmetric appearance and loss of symmetry may indicate pathology, symmetry evaluation on medical images is very important and has been routinely performed in diagnosis of diseases and pretreatment evaluation. Therefore, applying symmetry evaluation function to deep learning algorithms in interpreting medical images is essential, especially for the organs that have significant inter-individual variation but bilateral symmetry in a person, such as mastoid air cells. In this study, we developed a deep learning algorithm to detect bilateral mastoid abnormalities simultaneously on mastoid anterior–posterior (AP) views with symmetry evaluation. The developed algorithm showed better diagnostic performance in diagnosing mastoiditis on mastoid AP views than the algorithm trained by single-side mastoid radiographs without symmetry evaluation and similar to superior diagnostic performance to head and neck radiologists. The results of this study show the possibility of evaluating symmetry in medical images with deep learning algorithms.

## Introduction

With advancements of medical imaging techniques, the amount of medical images has increased greatly. Simple radiographs have been the most commonly performed medical imaging because of their cost-effectiveness and clinical usefulness^1,2^. This also leads to a huge increase in radiologists’ work-loads. Furthermore, as simple radiographs consist of complex three-dimensional anatomic information projected onto two-dimensional images, radiologists should have extensive medical and radiologic knowledge to interpret simple radiographs^2^.

Previous many studies have applied deep learning technology to interpretation of simple radiographs (e.g., chest X-ray, paranasal sinus radiographs, and mammography) to solve current clinical problems such as increased radiologists’ work-loads and great challenges of interpreting simple radiographs^3–7^. We also applied deep learning algorithms to interpret simple mastoid radiographs and showed promising results^8^. However, we found some limitations in this previous study. Because there were significant variations of the shape, size, and pneumatization pattern of mastoid air cells between individuals, it was difficult to diagnose mastoid disease using one unilateral mastoid view only. In comparison, the patterns of bilateral mastoid air cells in one person did not vary significantly. Therefore, symmetry evaluation using bilateral mastoid radiographs in one person is necessary to evaluate the presence of mastoiditis.

Symmetry is a very important feature of human anatomy, and loss of symmetry in medical images may indicate pathology^9^. This applies to not only organs that exist in pairs such as eyes, ears, breasts, kidneys, and limbs but also to organs with symmetric shapes including the brain, face, and vertebrae. Symmetry evaluation on medical images has been widely used in diagnosis of diseases, preoperative evaluation, and operative planning^10–13^. Previous studies reported that symmetry evaluation on chest radiography improved the ability of detecting pulmonary pathology^9,14^.

In this study, we developed a deep learning algorithm to detect bilateral mastoid abnormalities simultaneously on bilateral mastoid anterior–posterior (AP) views with symmetry evaluation. To assess the usefulness of the algorithm with symmetry evaluation, we compared the diagnostic performance of this algorithm with those of an algorithm detecting only single side abnormality without symmetry evaluation and head and neck radiologists.

## Methods

The Institutional Review Boards of Korea University Guro Hospital (KUGH) approved this study and waived informed consent considering the retrospective design and anonymized data used in this study. All the methods used in this study were performed in accordance with the relevant guidelines and regulations.

### Dataset

Mastoid series from 995 patients (1990 ears) were collected from KUGH between January 2018 and December 2020 to screen for mastoiditis. The mastoid series consisted of an AP view and two lateral views (i.e., left and right lateral views). Twenty-five patients with postoperative findings of mastoid air cells on either side were excluded. Among the remaining 1940 images from patients, 302 mastoid AP views of 151 patients who underwent temporal bone (TB) CT examinations within seven days of their mastoid series were used as the gold standard test set. The remaining 1638 mastoid AP views were used as the dataset for developing our deep learning algorithm. The training set and the validation set were generated by randomly dividing the whole dataset at 5:1 using stratified random sampling for symmetry of bilateral mastoiditis. We also collected temporal and geographic external test sets to further verify the performance of the deep learning algorithm. The temporal external test set was composed of 298 images from 149 patients in KUGH from January 2021 to May 2021. The geographic external test set was composed of 306 images from 153 patients in Korea University Anam Hospital from October 2018 to February 2019.

### Labeling

Digital Imaging and Communication in Medicine (DICOM) files of the mastoid series were downloaded from the picture archiving and communication system (PACS), and all the image data were anonymized for analyses.

Two head and neck neuroradiologists (I.R. and L.S., with 14 years and 13 years of experience in this field) independently labeled all mastoid AP views based on both AP lateral views, and the labels were determined by consensus. The training and validation sets (mastoid AP views of 819 patients, total images of 1638 ears) and the temporal and geographic external test sets were labeled based on the radiographic findings on mastoid AP and lateral views, whereas the gold standard test sets (302 mastoid AP views) were labeled based on the results of concurrent TB CT.

All the images in the training and validation sets and temporal and geographic external test sets were labeled in 4 categories: category 0, normal, clear mastoid air cells on both views; category 1, mild, some haziness of mastoid air cells on AP view or lateral view; category 2, severe, total haziness and sclerosis of mastoid air cells on both AP and lateral views; and category 3, mastoidectomy state. As postoperative states show inherently asymmetric appearance, images of category 3 could not be used in symmetry evaluation and were excluded. The gold standard test sets were labeled as category 0, normal; category 1, mild, soft tissue densities in some mastoid air cells; category 2, severe, soft tissue densities in most air cells with sclerosis; and category 3, postoperative state according to the results of TB CT. Again, images of category 3 in the gold standard test set were excluded.

For comparison of the diagnostic accuracy of the algorithm and the head and neck neuroradiologists, two head and neck neuroradiologists labeled the gold standard test set (images of 302 ears from 151 patients) based on only mastoid AP views according to the following criteria: category 0, normal, clear mastoid air cells; category 1, mild, some haziness of mastoid air cells, slightly increased density compared with contralateral clear mastoid air cells, or slightly decreased density compared with contralateral totally hazy mastoid air cells; and category 2, severe, total haziness and sclerosis of mastoid air cells; category 3, postoperative state.

We defined symmetry grade as the absolute difference of the mastoiditis category of the bilateral ears on the AP view. Symmetry was graded as grade 0, both ears have the same mastoiditis category, {right, left} mastoiditis is {normal, normal}, {mild, mild}, or {severe, severe} cases; grade 1, a one-stage difference between the two mastoiditis categories, i.e., {normal, mild}, {mild, severe} cases (vice versa); grade 2, a two-stage difference between the two mastoiditis categories, i.e., {normal, severe} cases (vice versa).

### Image preprocessing

The mastoid AP view shows both ears with mastoid air cells. We cropped the ears using the following method: the right ear was cropped at 180 mm × 120 mm centered on coordinates 0.5 and 0.25 times the original height and width of the AP view, respectively, and the left ear was cropped based on the symmetric point in the same way as the right ear. The cropped images were resized to 384 × 256 pixels, and the input image was composed by concatenating the two images in the vertical direction on the image plane. When concatenating the images, the left ear was flipped to the direction of the right ear. By unifying the direction, the two images had a high spatial relationship.

### Developing deep learning algorithms

**Figure 1:**
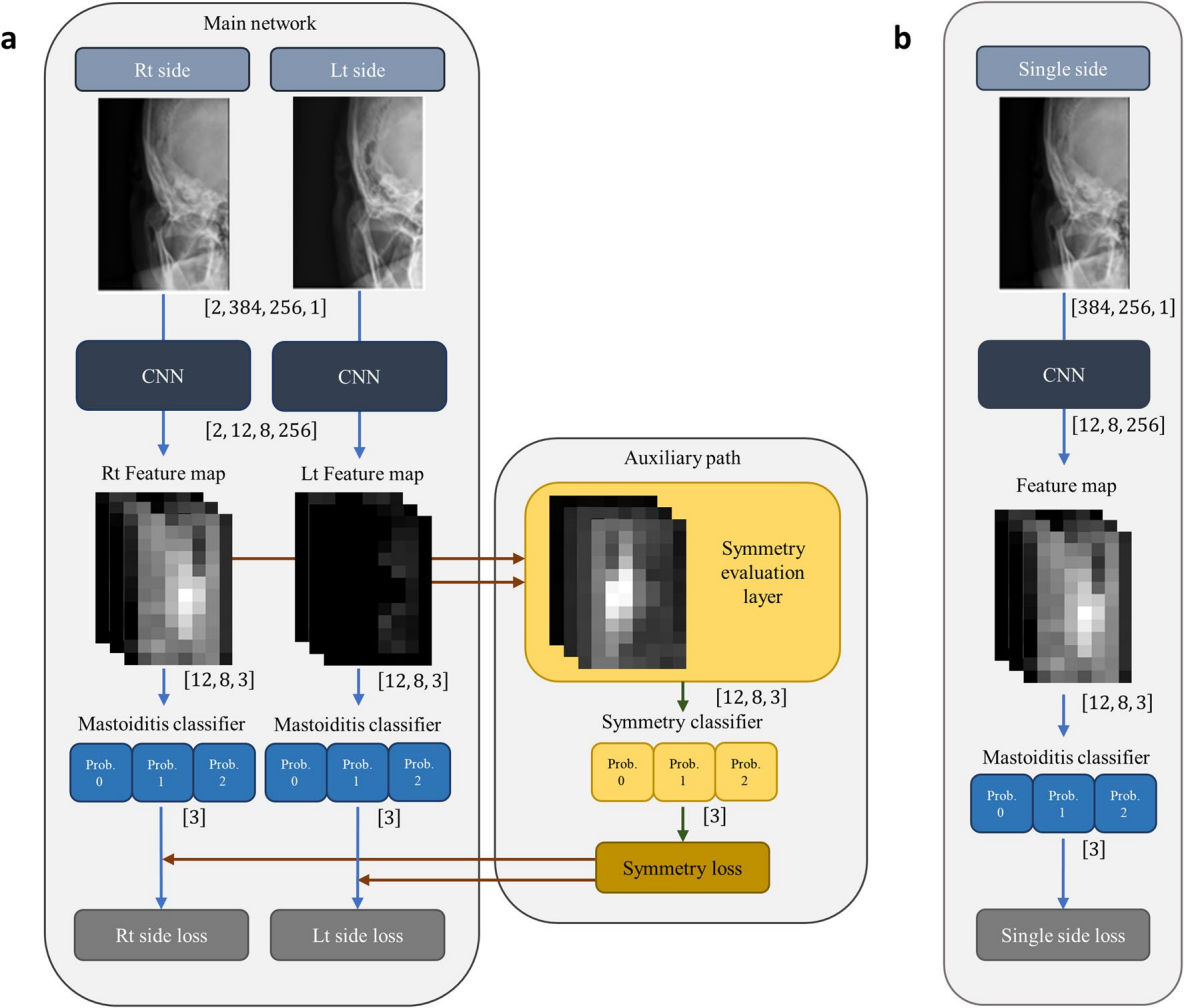
Network architectures of deep learning algorithms using both sides of mastoid anterior–posterior (AP) view with symmetry evaluation function and using single side. (**a**) A network architecture that predicts mastoiditis from both sides using symmetry evaluation. This architecture consists of a main network and an auxiliary path. The main network receives images of both ears as input, and three categories of mastoiditis are predicted through a convolutional neural network (CNN), feature map, and mastoiditis classifier for each ear. Each mastoiditis classifier predicts three mastoid categories as a probability vector. The symmetry evaluation layer in the auxiliary path receives both feature maps corresponding to the three mastoiditis categories and calculates the absolute value per pixel between both feature maps. The symmetry classifier predicts the difference between mastoiditis categories on both sides as three-valued probability vector. The symmetry loss is calculated from the symmetry classifier, and this loss is added to each mastoiditis loss calculated from the mastoiditis classifiers to obtain the final losses on both sides. (**b**) A network architecture predicting mastoiditis on only one side. This architecture receives only the image of one ear as input and predicts mastoiditis with similar structure to the main network in (**a**).

We designed a deep learning algorithm to predict mastoiditis by incorporating bilateral symmetry on the AP view (symmetry algorithm). The network architecture is shown in Fig. 1a and predicts bilateral mastoiditis with evaluating symmetry. Our architecture is composed of two components: a main network and an auxiliary path. The main network consists of two identical convolutional neural networks (CNNs) and mastoiditis classifiers. The two CNNs have the same weight to consider the relationship between two images with high spatial relation and performed convolution operations independently. Each CNN yields a feature map for mastoiditis prediction from each image and consists of six residual blocks^15^. The squeeze-and-excitation module was applied to each residual block^16^. The mastoiditis classifier predicts the degree of mastoiditis using the Softmax function after Log-Sum-Exp pooling on the feature map^17^. Second, the auxiliary path consists of a symmetry evaluation layer and a symmetry classifier. The symmetry evaluation layer receives both feature maps of the main network and evaluates the symmetry between these maps. Both feature maps in the main network are arrays with dimensions of $12\times 8\times 3$, respectively. The 12 and 8 represent reduced height and width, and the 3 represents three mastoiditis categories. The symmetry evaluation layer calculates the absolute value of the features of mastoiditis on both sides by grade and pixel. Finally, similar to the process of predicting mastoiditis on one side, the symmetry classifier predicts three levels of symmetry grade with Log-Sum-Exp pooling from the output that has passed through the symmetry evaluation layer.

To train using symmetry evaluation, the loss function was designed as follows. Three categorical cross-entropy losses were calculated for the mastoiditis classifier and the symmetry classifier. The symmetry loss calculated from the symmetry classifier was added to the loss of each mastoiditis classifier. Finally, the sum of losses of both mastoiditis classifiers was set for training. During training, the initial learning rate was set to 0.0001 using an RMSprop optimizer^18^. He initialization was used as the weight initialization^19^. Mini-batch size was set to 4.

We considered a network predicting mastoiditis for a single ear to compare with the symmetry algorithm (Fig. 1b). This network removed the path for predicting mastoiditis with symmetry of the contralateral ear from the network shown in Fig. 1a. Categorical cross-entropy loss was used as the loss function. During training, the optimizer, weight initialization, and mini-batch size were the same as for training of the symmetry algorithm. We trained networks on CUDA/cuDNN (version 10.1 and 7.0) and Tensorflow 2.3.0 with a Linux operating system. The OS, CPU, and GPU were Ubuntu 16.04, Intel^®^ Xeon^®^ Silver 4110 CPU @ 2.10 GHz, and GeForce RTX 2080 Ti, respectively.

We used class activation mapping to investigate the qualitative results of the symmetry algorithm^20^. The class activation mappings were computed for each of the three types of images: the right ear, the left ear, and the difference between the bilateral ears. The class activation mappings of the bilateral ears were computed from the right and left feature map in Fig. 1a for three mastoiditis categories (normal, mild, and severe). The right and left feature maps each consisted of 12 × 8 pixels with three channels matched to the three categories. Rectified Linear Unit (ReLU) activation was applied to highlight regions that strongly respond to the Log-Sum-Exp pooling calculation. These feature maps were resized to that of the original input for each channel. Next, we calculated the class activation mapping for the difference between ears to assure that the symmetry algorithm detected symmetry. An image of the difference between the ears was obtained from the symmetry evaluation layer. The feature maps for symmetry also consisted of 12 × 8 pixels with three channels, each for symmetry grades 0, 1, and 2. We computed class activation mapping of symmetry grades in the same way as that of mastoiditis categories.

### Statistical analysis

Area under the receiver operating characteristic (ROC) curve (AUC) was used as an evaluation metric of the deep learning algorithms and radiologist results. A 95% confidence interval (CI) of AUC was calculated by the method of DeLong et al. using two correlated ROC curves^21^. The three categories of unilateral mastoiditis were dichotomized as negative if normal and positive if mild or severe to calculate AUC. The prediction results of mastoiditis were dichotomized as follows: if the highest probability among predicted categories was normal, the prediction result was negative; if the probability was mild or severe, the prediction result was positive. Sensitivity and specificity were calculated as measures of diagnostic performance based on the prediction results. The 95% CI was calculated using Clopper–Pearson’s exact test^22^.

All statistical analyses were performed in R statistical software version 3.6.3 (The R Foundation for Statistical Computing, Vienna, Austria). A p-value less than 0.05 was considered statistically significant.

## Results

### Diagnostic performance analysis

**Figure 2:**
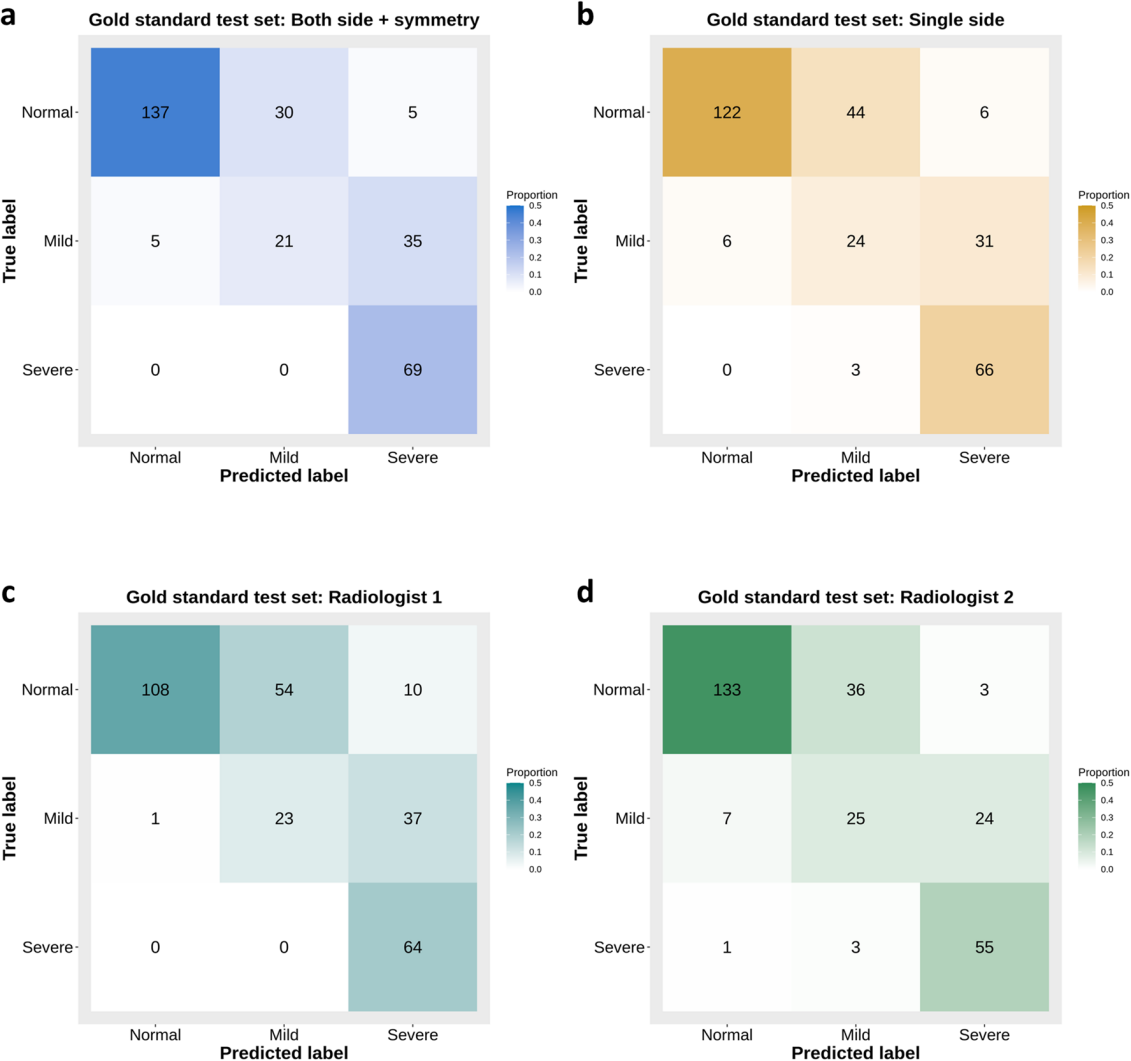
Confusion matrices for three mastoiditis categories with gold standard test set. (**a**) Confusion matrix of the algorithm using both sides of mastoid anterior–posterior (AP) view with symmetry evaluation function. (**b**) Confusion matrix of the algorithm using single side without symmetry evaluation. (**c**,**d**) Confusion matrices of two head and neck radiologists. The algorithm with symmetry evaluation shows higher accuracy for normal categories than the algorithm using single side and similar accuracy for mild and severe categories.

The baseline characteristics of the training set, validation set, gold standard test set, temporal external test set, and geographic external test set are shown in Table 1. The diagnostic performance of the algorithm predicting bilateral mastoiditis with symmetry evaluation (symmetry algorithm) was compared with those of an algorithm using a single side without symmetry evaluation and two radiologists using the gold standard test set, as shown in Table 2. The AUCs of the symmetry algorithm and algorithm using a single side were 0.879 (95% CI, 0.845–0.913) and 0.832 (95% CI, 0.793–0.870), respectively. The AUCs of radiologists 1 and 2 were 0.810 (95% CI, 0.773–0.847) and 0.856 (95% CI, 0.818–0.893), respectively. The AUC of the symmetry algorithm was significantly higher than the others except that of radiologist 2. The confusion matrices of deep learning algorithms and radiologists for three categories (normal, mild, and severe) of mastoiditis are shown in Fig. 2. The total accuracies of the symmetry algorithm and algorithm using a single side were 75.2% (95% CI, 69.9–80.0%) and 70.2% (95% CI, 64.7–75.3%), respectively (Fig. 2a,b). Ears predicted by radiologists to be in a mastoidectomy state were excluded from the confusion matrix. The total accuracies of radiologists 1 and 2 were 65.7% (95% CI, 60.0–71.0%) and 74.2% (95% CI, 68.7–79.2%), respectively (Fig. 2c,d).

**Table 1:** Baseline characteristics of all data sets.

Characteristic	Training set (n = 1370)	Validation set (n = 268)	Test set		
			Gold standard test set (n = 302)	Temporal external test set (n = 298)	Geographic external test set (n = 306)
Number of patients	685	134	151	149	153
Age (mean ± sd)	52.9 ± 12.8	52.8 ± 15.6	53.5 ± 16.7	54.6 ± 12.2	52.3 ± 13.4
Sex					
Female	305	65	77	65	67
Male	380	69	74	84	86
Label (based on CR)					
0, Normal	699	139		164	167
1, Abnormal	671	129		134	139
Mild	229	46		53	57
Severe	442	83		81	82
Label (based on CT)					
0, Normal			172		
1, Abnormal			130		
Mild			61		
Severe			69		

*CR* computed radiography including anterior posterior view and lateral view, *CT* computed tomography.

**Table 2:** Comparison of the diagnostic performance between the algorithm using both side with symmetry evaluation function, the algorithm using single side, and two radiologists in gold standard test set.

	Both side with symmetry	Single side		Radiologist 1		Radiologist 2	
	AUC	AUC	P*	AUC	P*	AUC	P*
Gold standard test set	0.879 (0.845–0.913)	0.832 (0.793–0.870)	0.001*	0.810 (0.773–0.847)	< 0.001*	0.856 (0.818–0.893)	0.247

Data is shown to three decimal places, with the 95% confidence interval in parentheses.

*AUC* area under the receiver operating characteristic (ROC) curves.

P*: P-value of one-side DeLong’s test for two correlated ROC curves (Alternative hypothesis: AUC of both side with symmetry was greater than AUC of single side or two radiologists).

*< 0.05 was significant.

The sensitivity and specificity of the two deep learning algorithms and radiologists are shown in Table 3. The sensitivity and specificity of the symmetry algorithm were 96.2% (95% CI, 91.3–98.7%) and 79.7% (95% CI, 72.9–85.4%), respectively, and those of the algorithm using a single side were 95.4% (95% CI, 90.2–98.3%) and 70.9% (95% CI, 63.5–77.6%). The sensitivity and specificity of radiologist 1 were 99.2% (95% CI, 95.8–100.0%) and 62.8% (95% CI, 55.1–70.0%), respectively, and those of radiologist 2 were 93.8% (95% CI, 88.2–97.3%) and 77.3% (95% CI, 70.3–83.4%).

**Table 3:** Comparison of the sensitivity and specificity for gold standard test set between the deep learning algorithms and head and neck radiologists based on the labels by standard reference (temporal bone CT).

	Sensitivity	*P*^*se*^	Specificity	*P*^*sp*^
Deep learning algorithm				
Both side with symmetry	96.2% (125/130, 91.3–98.7%)		79.7% (137/172, 72.9–85.4%)	
Single side	95.4% (124/130, 90.2–98.3%)	1.000	70.9% (122/172, 63.5–77.6%)	0.004*
Radiologist				
Radiologist 1	99.2% (129/130, 95.8–100.0%)	0.134	62.8% (108/172, 55.1–70.0%)	< 0.001*
Radiologist 2	93.8% (122/130, 88.2–97.3%)	0.546	77.3% (133/172, 70.3–83.4%)	0.571

Data are percentages and nominator/denominator, and 95% confidence interval in the parentheses.

*AUC* area under the receiver operating characteristic (ROC) curves.

*P*^*se*^, *P*^*sp*^: P values for comparing sensitivities/specificities between the deep learning algorithm and the radiologists were determined by using McNemar’s test.

*< 0.05 was significant.

### External validation

The diagnostic performances of deep learning algorithms were also evaluated with the temporal external test set and geographic external test set (Table 4). The AUCs of the symmetry algorithm and algorithm using a single side with the temporal external test set were 0.861 (95% CI, 0.822–0.899) and 0.815 (95% CI, 0.777–0.856), respectively. Those AUCs with the geographic external test set were 0.845 (95% CI, 0.807–0.884) and 0.817 (95% CI, 0.778–0.856), respectively.

**Table 4:** External validation of the diagnostic performance between the algorithm using both side with symmetry evaluation function and the algorithm using single side.

	Both side with symmetry	Single side	P*
	AUC	AUC	
Temporal test set	0.861 (0.822–0.899)	0.815 (0.777–0.856)	0.004*
Geographic test set	0.845 (0.807–0.884)	0.817 (0.778–0.856)	0.064

*: <0.05 was significant.

**Figure 3:**
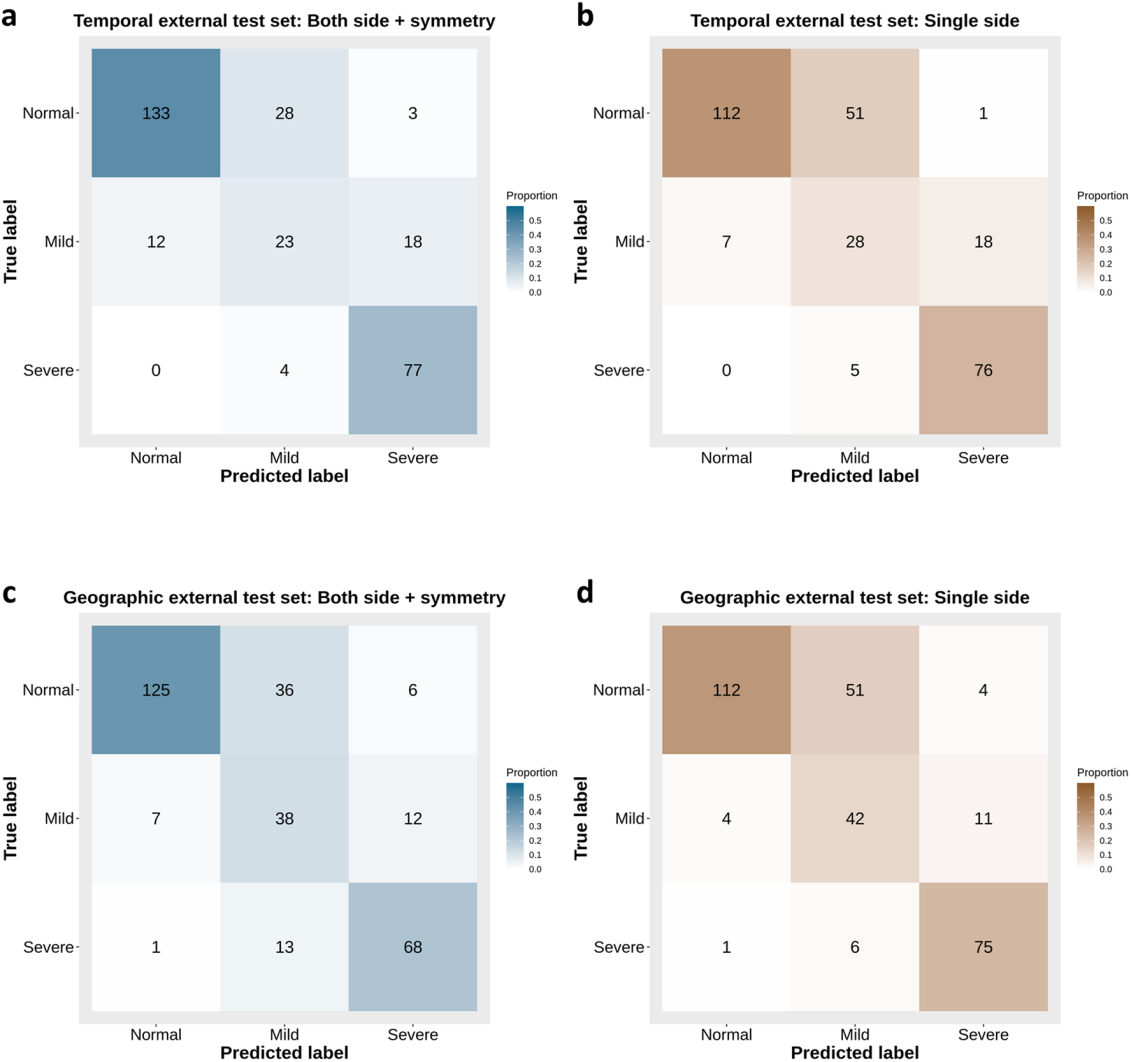
Confusion matrices for three mastoiditis categories predicted by deep learning algorithms with external validation sets. (**a**,**b**) Confusion matrices with temporal external test set. (**a**) Confusion matrix of the algorithm using both sides of mastoid anterior–posterior (AP) view with symmetry evaluation function. (**b**) Confusion matrix of the algorithm using single side without symmetry evaluation. (**c**,**d**) Confusion matrices with geographic external test set. (**c**) Confusion matrix of the algorithm using both sides of mastoid AP view with symmetry evaluation function. (**d**) Confusion matrix of the algorithm using single side without symmetry evaluation**.** The algorithm with symmetry evaluation has higher accuracy for normal categories than the algorithm using single side.

The confusion matrices of the algorithms with the two external test sets are shown in Fig. 3. In the temporal external test set, the total accuracy of the symmetry algorithm was 78.2% (95% CI, 73.1–82.7%), and that of the algorithm using a single side was 72.5% (95% CI, 67.0–77.5%). In the geographic external test set, the total accuracy of the symmetry algorithm was 75.4% (95% CI, 70.3–80.2%), and that of the algorithm using a single side was 74.8% (95% CI, 69.6–79.6%). The total accuracy of the symmetry algorithm was higher than that of the algorithm using a single side in both external test sets.

### Class activation mappings

**Figure 4:**
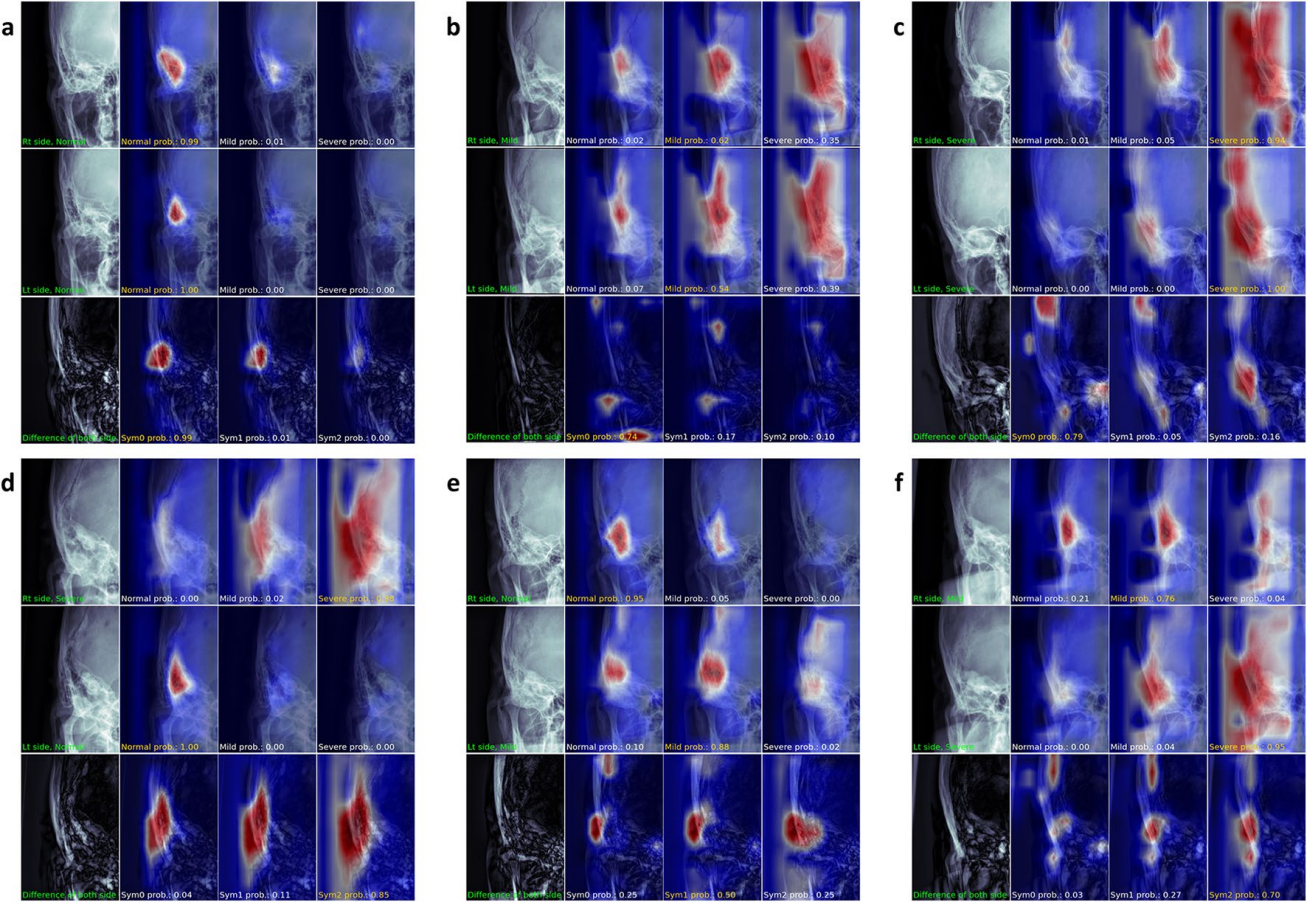
Class activation mappings from deep learning algorithm using both sides of mastoid anterior–posterior (AP) view with symmetry evaluation function. Each figure consists of 12 subfigures. The first and second rows in each image are the original mastoid AP view images and class activation mappings for normal, mild, and severe categories, respectively. The third row consists of an image showing the difference between the right and left ears and class activation mappings for symmetry grades 0, 1, and 2. Probabilities of mastoiditis category or symmetry grade are annotated on the corresponding class activation mapping. (**a**) Class activation mappings of bilateral normal mastoid air cells. (**b**,**c**) Class activation mappings of bilateral symmetric mild (**b**) and severe (**c**) mastoiditis cases. (**d**) Class activation mappings of a case with right side severe mastoiditis and left side normal mastoid air cells (symmetry grade 2) (**e**,**f**) Class activation mappings of symmetry grade 1 cases (**e**) a case with right side normal mastoid air cells and left side mild mastoiditis and (**f**) a case with right side mild mastoiditis and left side severe mastoiditis.

The class activation mappings for all possible combinations of mastoiditis categories and symmetry grades using the symmetry algorithm are shown in Fig. 4. Probabilities of mastoiditis category or symmetry grade are annotated on the corresponding class activation mapping. In class activation mapping, strongly reactive regions for each grade are red, intermediate regions are white, and unreacted regions are blue. For bilateral normal cases, the algorithm strongly detects regions of mastoid air cells without mastoiditis or normal mastoid air cells in both ears, and the class activation mapping of symmetry grade 0 showed greater detection of normal mastoid air cells compared to the other two class (symmetry grade 1 and 2) activation mappings (Fig. 4a). For bilateral symmetric mild or severe mastoiditis, the algorithm assessed a wider range around mastoid air cells (Fig. 4b,c). Since the characteristics of normal mastoid air cells disappear as mastoiditis severity increases, the algorithm assesses the mastoiditis category over a wider region than the mastoid air cells (Fig. 4b,c). The class activation mappings of symmetry evaluation predict a symmetry grade as 0 because normal mastoid air cells are not detected on both sides (Fig. 4b,c). For symmetry grade 2 cases, the algorithm detects normal mastoid air cells on one side and no mastoid air cells on the other side. Therefore, the algorithm can detect the difference between bilateral mastoid air cells clearly (Fig. 4d). However, for cases with symmetry grade 1, the algorithm detects some areas of mastoid air cells in mild mastoiditis (Fig. 4e,f). Therefore, the prediction power of symmetry grade is worse than that for cases with symmetry grade 2, especially for those with bilateral mastoiditis (mild on one side and severe on the other side (Fig. 4f).

## Discussion

Symmetry is a very important anatomic feature in interpreting human medical images because not only many organs such as eyes, ears, salivary glands, breasts, kidneys, limbs, and even vascular structures such as carotid arteries exist in bilateral paired structures but also many other single organs including face, brain, thyroid gland, and axial bones have symmetric appearance. Evaluation of symmetry on medical images has been performed to diagnose pathology or to plan orthopedic and plastic surgeries^10–13^. Symmetry evaluation is especially useful for interpreting regions of bilateral symmetry in one person but large anatomic variation between individuals. Mastoid air cells have significant anatomic variations in size, shape, and pneumatization pattern between and within individuals, as the shape of mastoid air cells changes greatly during age^23–26^. However, bilateral mastoid air cells show little anatomic variation in one person at any certain time. Therefore, it is very important to evaluate symmetry to diagnose mastoid disease on mastoid views.

In the present study, we trained an algorithm using bilateral mastoid AP views to aid in symmetry evaluation in the diagnosis of mastoiditis. For symmetry evaluation, we graded the concordance between the mastoiditis categories of the bilateral ears. The symmetry algorithm predicted mastoiditis categories by incorporating symmetry evaluation based on grade concordance with the contralateral ear. The symmetry algorithm showed better performance than the algorithm trained with a single-side AP view. Furthermore, the symmetry algorithm showed similar or superior performance to head and neck radiologists.

Our algorithm evaluated symmetry by simply calculating the pixel-by-pixel difference between the feature maps extracted from bilateral ears. There have been several attempts to automatically detect malignant lesions by adding symmetry information in medical imaging such as mammography^27,28^. These approaches concatenated the network of the main image by adding the network of the contralateral image in parallel. To incorporate symmetry information more intuitively, we added a layer that evaluates symmetry between two parallel CNN streams.

Interestingly, the class activation maps of the algorithm trained by bilateral mastoid AP views simultaneously detected normal mastoid air cells in the present study, whereas the class activation maps of the previous study to diagnose mastoiditis on single-side view detected hazy, diseased mastoid air cells^8^. In the present study, if the symmetry algorithm detected bilateral normal mastoid air cells or did not detect bilateral mastoid air cells at all, the algorithm interpreted these findings as symmetric; if the algorithm detected only unilateral mastoid air cells, it interpreted that as asymmetric. However, since only some mastoid air cells were detected in the mild mastoiditis (category 1), the accuracy or prediction power in cases with mild (category 1) mastoiditis was worse than that of the cases of symmetry grade 0 or 2. This same tendency was observed with the radiologists’ daily practices.

As we mentioned earlier, as most human anatomical structures have symmetric appearance or exist in pairs, a deep learning algorithm to interpret medical images should also have the ability to evaluate the symmetry of structures and to apply those results to interpretation of the images to increase the diagnostic power. The results of this study show the possibility of evaluating symmetry in medical images with deep learning algorithms. Based on these results, symmetry evaluation can be adapted and studied in other research of human diseases involving anatomic regions with symmetric appearance.

There are some limitations in this study. First, mastoid series consist of AP and lateral views. However, due to the complexity of developing and applying a symmetry algorithm using both views together, we only used AP views. Therefore, the accuracy was somewhat inferior to that of the previous study using AP view and lateral view together. Development of algorithms with symmetry evaluation using multiple views will increase the accuracy. Second, only the gold standard test set had the actual status of mastoiditis based on TB CT. Other data sets did not have TB CT results. Even though the results of the geographic external test set showed higher accuracy for the symmetry algorithm than the algorithm trained with a single side, statistical significance was not achieved. This also could be explained by lack of TB CT results for the external test sets and less accurate results compared with the gold standard test set. Last, we developed the symmetry algorithm under the assumption that the mastoid air cells of bilateral ears overlap when the AP view is folded along the vertical bisector. If the original AP view had severe asymmetry, the accuracy of symmetry evaluation might be reduced.

## Conclusion

This study shows better performance of an algorithm with symmetry evaluation function using bilateral mastoid AP views than an algorithm trained using single-side images in diagnosing mastoiditis and similar or superior performance of the symmetry algorithm to head and neck radiologists. Since the most human organs exist in pairs or have symmetric appearance, symmetry evaluation on medical images is very important and has been routinely performed. Therefore, addition of symmetry evaluation function to deep learning algorithms for interpreting medical images is essential, and this study shows a promising result to applying that function. Further studies with multiple image views together are necessary to increase the accuracy of the algorithm.

## Data Availability

The data sets generated and analysed in this study are available from corresponding authors (IR and KJL) on reasonable request.
